# Intravoxel incoherent motion imaging and dynamic susceptibility contrast perfusion MRI in differentiation between recurrent intracranial tumor and treatment-induced changes

**DOI:** 10.1007/s00234-025-03575-4

**Published:** 2025-03-21

**Authors:** Jussi Hellström, Ishita Huq, Petra Witt Nyström, Erik Blomquist, Sylwia Libard, Raili Raininko, Johan Wikström

**Affiliations:** 1 Section of Neuroradiology, Department of Surgical Sciences, Uppsala University, Uppsala, Sweden; 2 Department of Immunology, Genetics and Pathology, Uppsala University, Uppsala, Sweden; 3 Department of Oncology, Uppsala University, Uppsala, Sweden; 4 Department of Surgical Pathology, Uppsala University Hospital, Uppsala, Sweden; 5 Department of Surgical Sciences, Uppsala University, Uppsala, Sweden

**Keywords:** Intracranial neoplasms, Tumor recurrence, Radiation injuries, Magnetic resonance imaging, Perfusion

## Abstract

**Purpose:**

To compare intravoxel incoherent motion (IVIM) imaging to dynamic susceptibility-weighted contrast (DSC) perfusion MRI in differentiating tumor recurrence from treatment-induced changes.

**Methods:**

Our prospective study included patients previously treated with radiotherapy for intracranial tumors who later developed a new or increasing contrast-enhancing lesion. The final diagnosis was based on neuropathology or 6-month follow-up. MR examinations were performed for calculation of the perfusion fraction (f) using the IVIM technique and relative blood volume (rCBV) using DSC perfusion. Measurements of f and rCBV were made by two independent readers in hotspots when possible, but otherwise in the whole enhancing region. Measures of rCBV were normalized to the contralateral region. Receiver operating characteristics (ROC) analysis was performed.

**Results:**

Sixty patients (35 men, median age 49, range 20–77) were evaluated. Forty-four patients had tumor recurrence and 16 had treatment-induced changes. Mean f was 0.090 for tumors and 0.058 for treatment-induced changes (*p* = 0.002). Mean rCBV was 3.52 and 1.79, respectively (*p* = 0.002). The area under the curve (AUC) in the ROC analysis was 0.72 for f and 0.77 for rCBV. Cutoff values of 0.073 for f and 2.26 for rCBV yielded equal values for sensitivity (73%), specificity (75%), and accuracy (73%). The 90th percentile value of rCBV was 4.77 for tumors and 2.53 for treatment-induced changes (*p* = 0.0004) and yielded the highest AUC (0.79) and a sensitivity/specificity/accuracy of 80%/75%/78% at cutoff value 3.25.

**Conclusion:**

The accuracy of the IVIM parameter f is similar to that of rCBV in differentiating tumor recurrence from treatment-induced changes.

**Supplementary Information:**

The online version contains supplementary material available at 10.1007/s00234-025-03575-4.

## Introduction

Differentiation between recurrent tumor and contrast-enhancing treatment effects is often difficult in patients given radiation therapy after resection of gliomas [1]. Treatment-related changes have been reported to be more frequent in combination with temozolomide [2]. A commonly used technique for the differentiation between tumor recurrence and treatment effects is dynamic susceptibility-weighted contrast (DSC) perfusion MRI with measurement of the relative cerebral blood volume (rCBV) [3].

Intravoxel incoherent motion (IVIM) imaging was introduced by le Bihan et al. in the 1980s [4, 5] and is based on a diffusion-weighted sequence with multiple b-values that permits separation of diffusion and perfusion effects [6]. At higher b-values (stronger motion sensitizing gradients), the signal loss comes primarily from diffusion effects, whereas at lower b-values (weaker motion sensitizing gradients), the microcirculation of blood in capillaries (i.e., perfusion) also contributes to signal loss. The signal loss can therefore be described via a bi-exponential model taking both perfusion and diffusion effects into account [7] for different b-values via the Eqs. [6, 8]

$$\:\frac{S}{{S}_{0}}={(1-f)e}^{-bD}+{fe}^{-b({D}^{*}+D)}$$

where S is the signal intensity, S_0_ signal intensity at baseline without motion sensitizing gradients, f is the perfusion fraction (volume of voxel occupied by capillaries), D is the water diffusion coefficient (which characterizes the diffusion of water molecules in the extravascular space), and D* is the pseudodiffusion coefficient (reflecting dephasing from perfusion in capillaries which are assumed to be organized randomly).

Some advantages of the IVIM method are that it allows simultaneous registration of diffusion and perfusion parameters and that no intravenous contrast agent needs to be injected. The IVIM method has regained interest in recent years particularly in the oncology field, but the lack of standardized image acquisition and analysis calls for further studies to investigate clinical applications [9]. There are only three earlier studies on the use of IVIM to differentiate tumor recurrence from contrast-enhancing treatment-induced changes [10–12] and the number of the subjects studied is still limited. The entire contrast-enhancing area was used in measurements in those studies, but using hotspots when such are found may be beneficial in the evaluation of heterogeneous lesions.

The aim of our prospective study was to compare IVIM to DSC perfusion MRI in differentiating tumor recurrence from treatment-induced changes, focusing measurements in the area with the highest perfusion or blood volume.

## Materials and methods

### Study population

The study protocol was approved by the National Ethics Committee. All patients gave their written informed consent to participate in the study. The study was performed in accordance with the 1964 Helsinki declaration and its later amendments or comparable ethical standards.

**Fig. 1:**
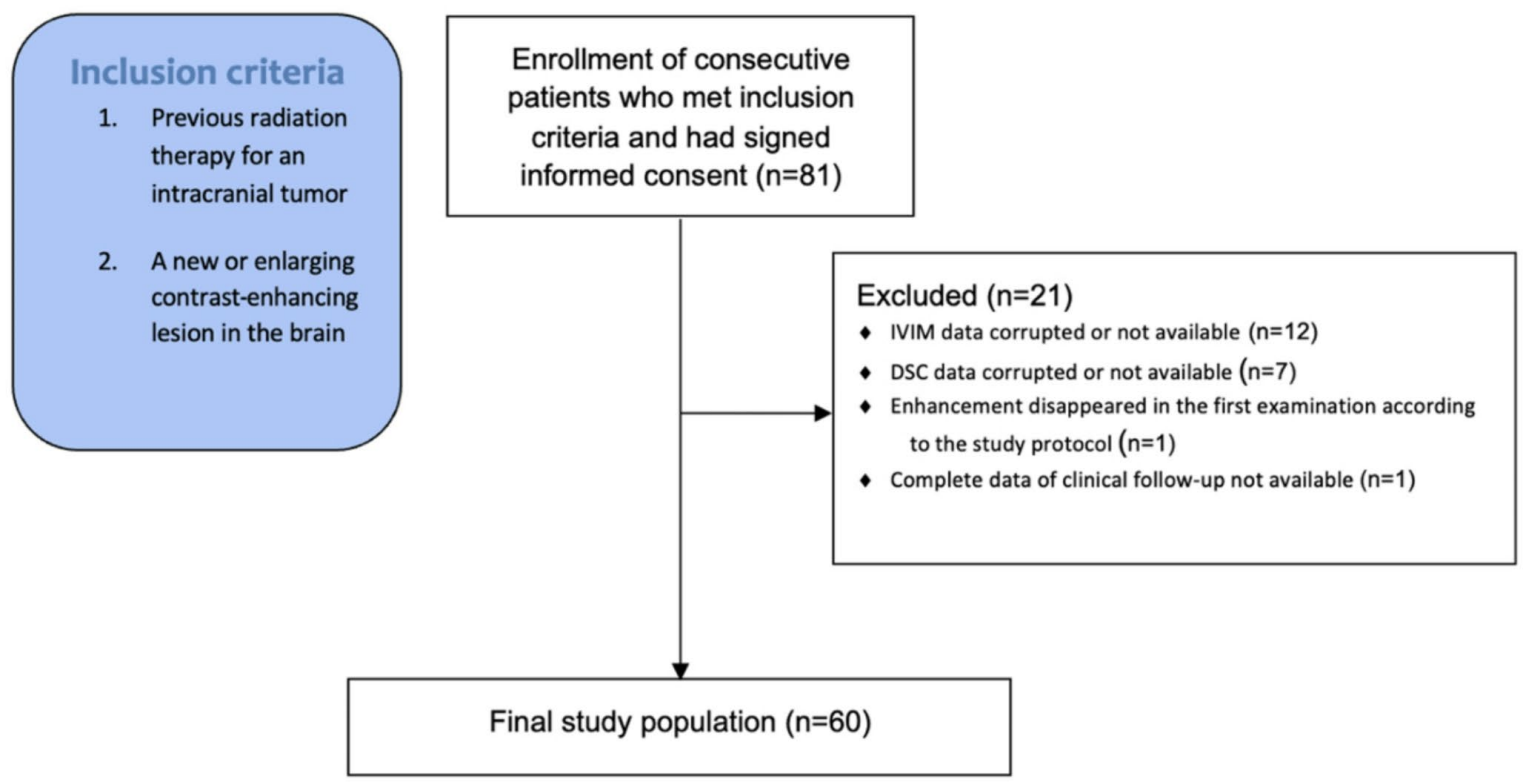
Flowchart describing inclusion and exclusion of the patients. DSC = Dynamic susceptibility-weighted contrast, IVIM = intravoxel incoherent motion

In this prospective study, 81 patients were consecutively included during a period of 6 years and 3 months. Inclusion criteria consisted of previous radiation therapy for an intracranial tumor and demonstration of a new or enlarging contrast-enhancing lesion in the irradiated region. Exclusion criteria consisted of missing/corrupt IVIM or DSC perfusion data, total regression of the lesion at the first examination according to the study protocol, and incomplete clinical follow-up data. See Fig. 1 for further information. Three patients were included twice because of the appearance of a new lesion matching the inclusion criteria.

### Imaging protocol

The patients underwent routine follow-up MRI examinations, and when a lesion matching the inclusion criteria appeared, the patients underwent an additional MRI examination using a dedicated imaging study protocol. MR imaging was performed on an Avanto 1.5T system (Siemens Medical Systems). The scanning protocol included axial diffusion-weighted images with b-values: 0, 50, 100, 150, 200, and 1000 s/mm^2^. The diffusion-weighted images were obtained with TR/TE 4700/89 ms, 5 mm slice thickness, in-plane resolution 1.2 × 1.2 mm, and 4 signal averages. For the diffusion-weighted images a spin echo echo planar imaging sequence was used. Axial T1-weighted image series (2D spin echo) were obtained with TR/TE 593/9.1 ms, 5 mm slice thickness, 0.6 × 0.6 mm in-plane resolution, and one signal average. The gadolinium contrast agent gadobutrol (Gadovist (Bayer)) of 0.1 mmol/kg body weight was injected intravenously at a rate of 5 ml /s followed by a saline flush of 30 ml at the same flow rate. A DSC perfusion study was performed with TR/TE 1410/30 ms, 5 mm slice thickness, 1.8 × 1.8 mm in-plane resolution and one signal average. Then an axial T1-weighted image series was obtained using the same parameters as before the gadolinium injection. The flip angle for diffusion-weighted imaging, DSC and T1 spin echo was 90 degrees.

### Image processing

The diffusion trace images and raw DSC perfusion data were imported into Olea Sphere 3.0 (Olea Medical). Computation of the diffusion parameters from the multi-b acquisition was performed via a Bayesian method [13], resulting in maps of the perfusion fractions f, D, and D*. DSC perfusion data were used to produce CBV maps with leakage correction.

### Image analysis

**Fig. 2:**
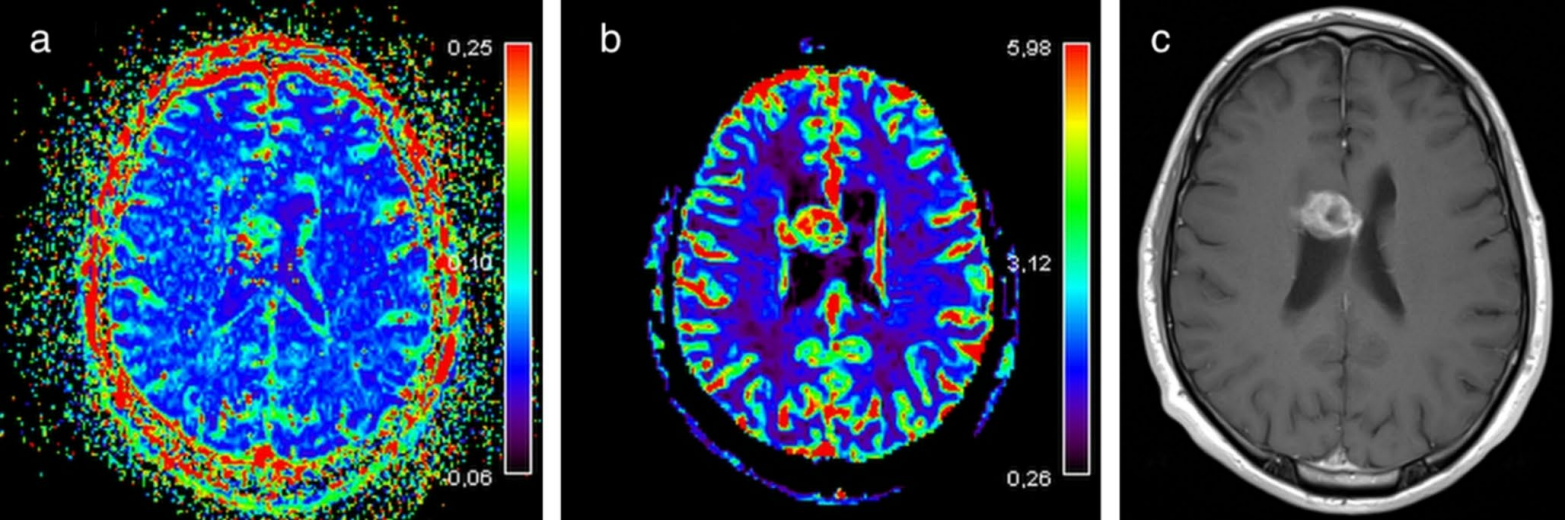
Recurrent tumor, neuropathologically confirmed ependymoma grade III. The f map (**a**) and rCBV map (**b**) show visual increase in their respective values in the lesion located close to the midline. The co-registered contrast-enhanced T1-weighted image (**c**) was overlaid on the perfusion maps when measurements were made

Measurements were made in perfusion hotspots on both f and rCBV maps if possible; otherwise, the entire contrast-enhancing area of the lesion was included in the drawn region of interest (ROI). A hotspot was defined as an area with higher perfusion values than the rest of the lesion provided that the higher values could not be attributed to noise. Care was taken not to perform measurements in areas of susceptibility artefacts. ROIs were drawn by free-hand and comprised at least 7 pixels. Some months before the final measurements were made, 10 randomly selected cases were reviewed together by the two readers (JH, IH) to set a standard for hotspot interpretation. The final measurements were performed independently by the two readers blinded to the final diagnosis. For reader 1 the mean area for f was 13.1 mm^2^ in hotspots (range 10.1–23.0 mm^2^) and the mean area for rCBV was 31.2 mm^2^ in hotspots (range 12.9-177.6 mm^2^). The second reader (IH) performed the measurements twice for each lesion, totaling three measurements for each lesion. The mean values of these three measurements are reported, and the measures of CBV were normalized to normal appearing contralateral brain parenchyma in a symmetrical ROI. Most often that implicated a placement in normal-appearing white matter because the tumors most often originated from white matter. The time between the measurements for reader 2 was three months for the majority of the cases. All cases were evaluated by both readers in the same consecutive order based on the inclusion time. In the post-processing software, the IVIM f parameter maps and rCBV maps were overlaid the co-registered contrast-enhanced T1-weighted images to enable measurements in the area of contrast enhancement. The same procedure was performed both with hotspots and measurements of the entire contrast-enhancing area of the lesion. Pixels with IVIM parameter f values > 0.3 were omitted via a threshold function to not include non-physiological values. An example of f and rCBV maps together with a contrast-enhanced T1-weighted image is given in Fig. 2.

### Neuropathology

The surgical specimens from the patients were identified in the database of the Department of Surgical Pathology, *Anonymized* University Hospital. Samples from the primary tumors were found in 54/60 subjects. Samples from suspected recurrences had been taken in 19 subjects, but tissue could not be found in one case. All available samples were reassessed by a neuropathologist. The samples were re-classified, and a final diagnosis was given according to 2021 WHO classification of tumors of the central nervous system [14]. Molecular analysis and immunohistochemical analysis were performed in accordance with standard procedures. If the samples were not available for reassessment, data from the original neuropathological evaluation was used.

### Final diagnosis

Neuropathological diagnosis was obtained if the lesion was biopsied or resected, and was then used as the final diagnosis. In cases where diagnostic tissue sample was lacking, the clinical diagnosis 6 months after study inclusion was used as the final diagnosis. During the follow-up period, at least one additional MRI examination was performed. The diagnosis was made by an oncologist, taking into account the clinical status of the patient and results of performed radiological examinations.

### Statistical methods

Calculations were made in R studio version 1.4.1717 with R version 4.1.0.

The correlation between f and rCBV was assessed by Pearson’s r coefficient (estimate and 95% confidence interval (95% CI) reported).

The level of agreement regarding whether a hotspot was present or not was assessed by Cohen’s kappa.

Intraclass correlation coefficients (ICCs) (two-way random effects, absolute agreement, single measurement) were reported with an estimate and CI for the inter- and intraobserver agreement of mean values f and rCBV.

Welch’s t-test was used to compare f, D*, and D values in tumor recurrence and treatment-induced changes.

Mann-Whitney U test was used for rCBV values when comparing tumor recurrence and treatment-induced changes (a non-parametric test was used because data were not normally distributed).

In the receiver operating characteristics (ROC) analysis, cutoff points were calculated by maximizing the sum of the sensitivity and specificity.

## Results

### Final study population

After exclusions described in the flowchart in Fig. 1, a total of 60 study subjects (median age was 49, range 20–77) were included in the study. The median radiation dose was 60 Gy (range 34–60), but for eight patients the dose was uncertain, e.g., patients treated with gamma knife and those with descriptions like “full dose” in the medical charts. Additional background data is provided in Table 1.

**Table 1:** Background data on the study subjects

Variable		No of subjects
Sex	Male	35
	Female	25
Radiation therapy	Photon	54
	Photon + Proton	3
	Gamma knife	2
	Proton	1
Time between radiation therapy and inclusion in the study	< 6 months	13
	6–12 months	9
	1–3 years	24
	> 3 years	14
Type of irradiated tumor	Glioblastoma, IDH wild type, grade 4 ^a^	29
	Astrocytoma, IDH mutant, grade 4	6
	Astrocytoma, IDH mutant, grade 2	6
	Oligodendroglioma, grade 3	5
	Astrocytoma, IDH mutant, grade 3 ^b^	4
	Oligodendroglioma, grade 2	2
	Metastasis	2
	Supratentorial ependymoma, grade 3	2
	Ganglioglioma, grade 3 ^a^	1
	Meningioma, grade 2	1
	Oligoastrocytoma grade 2 ^a^	1
	Oligoastrocytoma grade 3 ^a^	1
Chemotherapy before inclusion	Therapy including temozolomide	33
	Therapy not including temozolomide	6
	Uncertain if temozolomide was included ^c^	6
	No chemotherapy	15

^a^ For a single patient tissue was not available for re-examination

^b^ For two patients tissue was not available for re-examination

^c^ All medical charts from other hospitals not available. IDH = isocitrate dehydrogenase

Forty-four patients had tumor recurrence and 16 had treatment-induced changes. The 16 patients with treatment-induced changes had a primary World Health Organization tumor grade 1–2 in 4 cases and grade 3–4 tumor or metastasis in 12 cases. Nineteen of the diagnoses were made from neuropathological specimens: 18 recurrent tumors and one showing treatment-induced changes. The neuropathological specimen diagnoses are summarized in Table 2.

**Table 2:** Distribution of diagnoses made from neuropathological specimens in the present study compared to the diagnosis of the original tumor

Neuropathological diagnosis of the original tumor	Neuropathological diagnosis of the new contrast-enhancing lesion	No. of cases
Glioblastoma, IDH wild type, grade 4	Glioblastoma, IDH wild type, grade 4	9
Oligodendroglioma, grade 2	Oligodendroglioma, grade 3	2
Oligodendroglioma, grade 3	Oligodendroglioma, grade 3	1
Astrocytoma, IDH mutant, grade 2	Astrocytoma, IDH mutant, grade 4	1
Astrocytoma, IDH mutant, grade 3	Astrocytoma, IDH mutant, grade 3	1
Astrocytoma, IDH mutant, grade 4	Astrocytoma, IDH mutant, grade 4	1
Oligoastrocytoma, grade 2 ^a^	Glioblastoma grade 4 ^a^	1
Supratentorial ependymoma, grade 3	Supratentorial ependymoma, grade 3	1
Oligoastrocytoma, grade 3 ^a^	Glioblastoma, IDH wild type, grade 4	1
Glioblastoma, IDH wild type, grade 4 ^a^	Treatment-induced changes	1

^a^Tissue not available for re-examination. IDH = isocitrate dehydrogenase

### Inter- and intraobserver agreement

#### F maps

A hotspot was found by reader 1 in 30 cases and by the first read of reader 2 in 32 cases. There was an agreement on whether a hotspot for f was present or not in 80% (48/60) of cases between reader 1 and the first read from reader 2, yielding a Cohen’s kappa value of 0.6, representing moderate agreement. There was an agreement on whether a hotspot for f was present or not in 67% (40/60) of cases between the first and second reads from reader 2, yielding a Cohen’s kappa value of 0.34, representing fair agreement.

ICC for the mean value for f was 0.74 (95% CI 0.60–0.84) between reader 1 and the first observation of reader 2 and 0.59 (95% CI 0.40–0.73) between reader 1 and the second observation of reader 2. Thus, there was moderate reliability between the two readers. For the intraobserver agreement for reader 2, ICC was 0.59 (95% CI 0.37–0.74), also showing moderate reliability.

#### rCBV maps

A hotspot was found by reader 1 in 32 cases and by the first read of reader 2 in 36 cases. There was agreement on whether a hotspot for rCBV was present or not in 77% (46/60) of cases between reader 1 and first read of reader 2, yielding a Cohen’s kappa value of 0.53, representing moderate agreement. There was agreement on whether a hotspot for rCBV was present or not in 67% (40/60) of cases between first and second read by reader 2, yielding a Cohen’s kappa value of 0.34, representing fair agreement.

ICC for the mean value for rCBV was 0.88 (95% CI 0.77–0.94) between reader 1 and the first observation of reader 2, and 0.81 (95% CI 0.71–0.88) between reader 1 and the second observation of reader 2. The ICC for the intraobserver agreement for reader 2 was 0.85 (95% CI 0.75–0.91). The results for ICC regarding rCBV measurement indicated good reliability.

### Correlation between f and rCBV

**Fig. 3:**
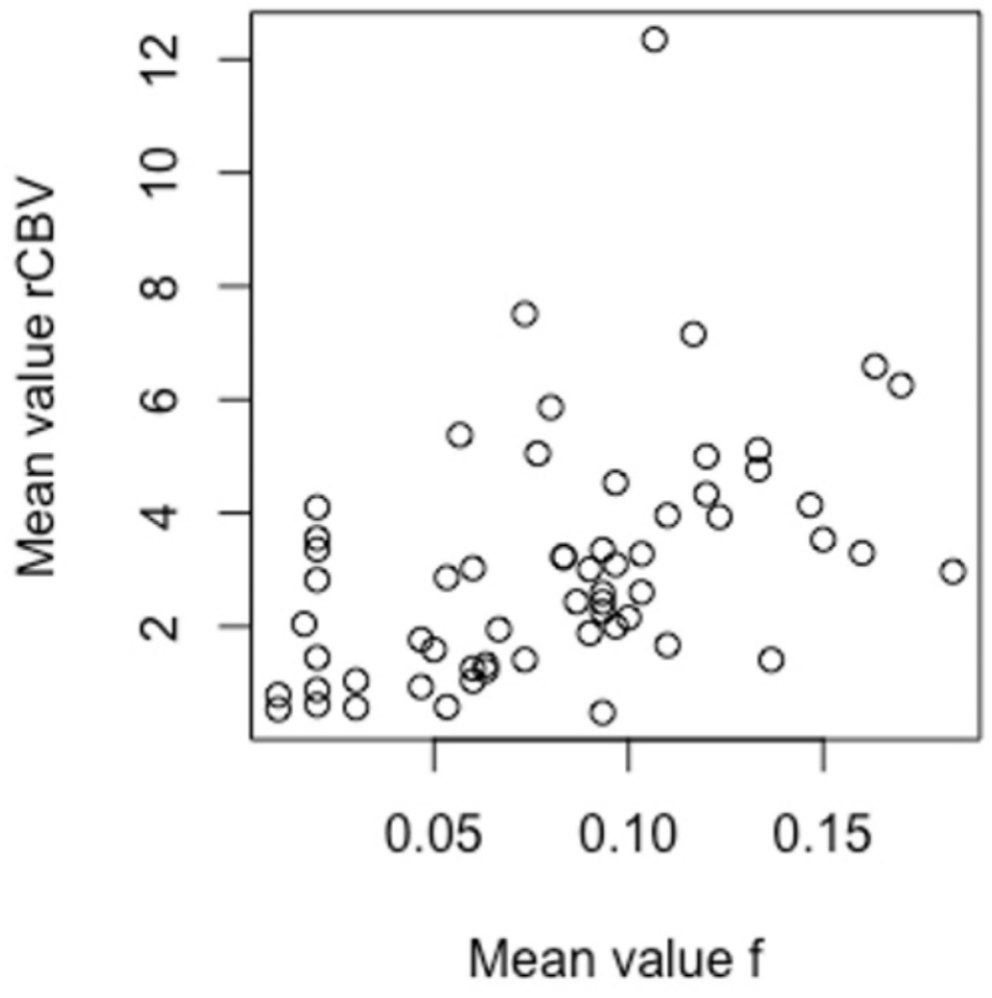
Scatterplot of mean values of f and rCBV. Pearson’s r coefficient was 0.45 when the outlier was included and 0.50 when it was excluded. f = perfusion fraction, rCBV = relative cerebral blood volume

Pearson’s r coefficient was 0.45 (95% CI 0.22–0.63, *p* = 0.0003) for the correlation between the mean values of f and rCBV (Fig. 3). With the outlier (the highest rCBV value) excluded, Pearson’s r coefficient was 0.50 (95% CI 0.28–0.67, *p* = 0.00006). These values correspond to a moderate correlation, with and without the outlier.

### Comparison of perfusion parameter values

**Fig. 4:**
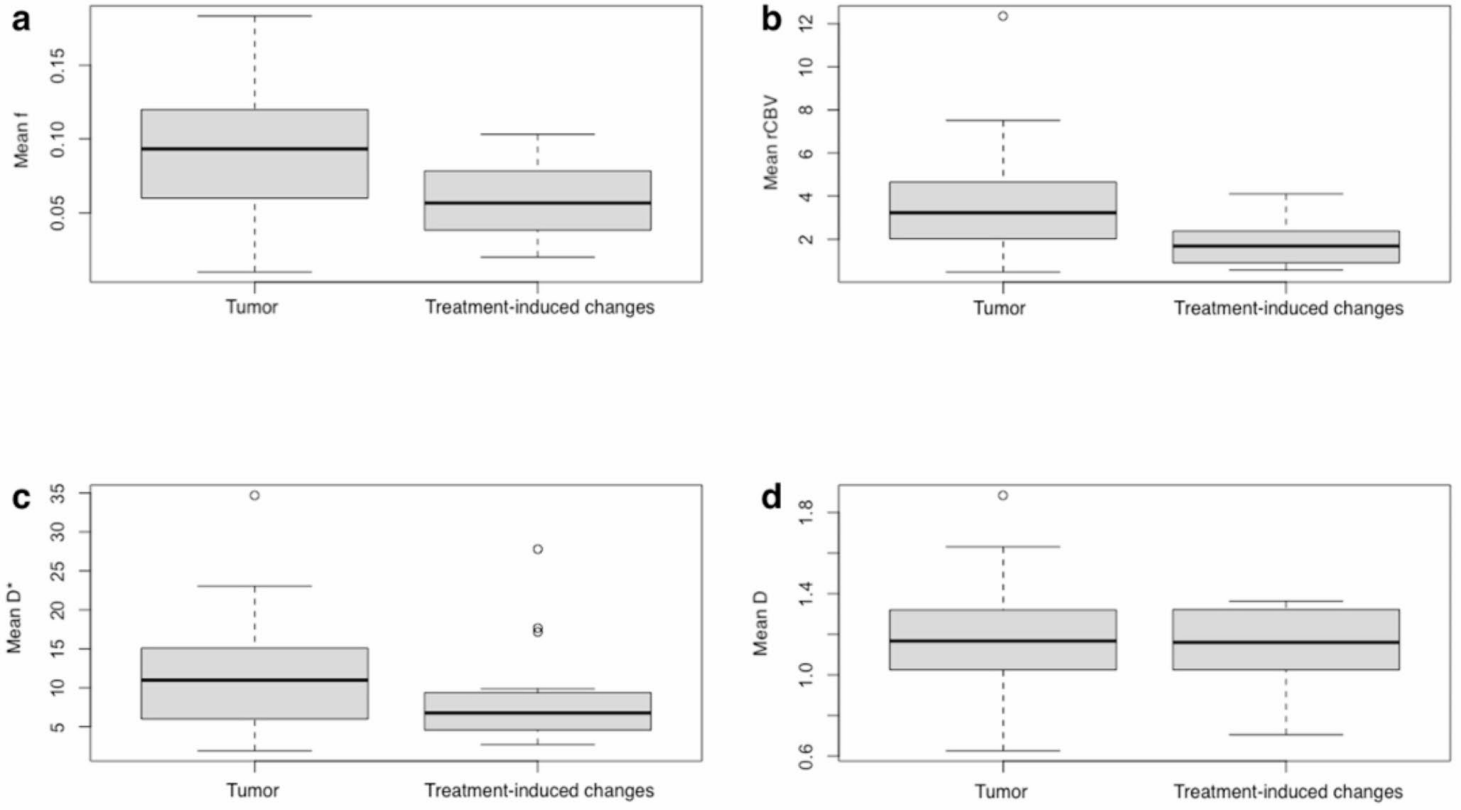
Box and whisker plots for mean values of f (**a**), rCBV (**b**), D* (**c**), and D (**d**) in recurrent tumors and treatment-induced changes. The perfusion measures are higher in the tumor group (*p* = 0.002 for f, *p* = 0.002 for rCBV). There are no significant diffusion differences between the tumors and treatment-induced changes. The horizontal line in the box represents the median value. The whiskers represent values within 1.5 x interquartile range value. A circle represents an outlier. D* and D are in units of x 10^− 3^ mm^2^/s D = water diffusion coefficient, D* = pseudodiffusion coefficient, f = perfusion fraction, rCBV = relative cerebral blood volume

Results of IVIM and DSC perfusion measurements in recurrent tumors and treatment-induced changes are reported in Table 3; Fig. 4. Mean f was 0.090 for tumors and 0.058 for treatment-induced changes (*p* = 0.002) (Table 3). Mean normalized rCBV was 3.52 for tumors and 1.79 for treatment-induced changes (*p* = 0.002). There were no significant differences in D* or D between tumors and treatment-induced changes. Figure 4a–d also reveal that although there was a highly significant difference in the f and rCBV values between the groups of the recurrent tumors and treatment-induced changes, there was a marked overlap between these two groups with all imaging methods. Additional data comparing recurrent tumors when the primary tumor had been glioblastoma (the largest subgroup) or another tumor can be found in Online Resource 1.

**Table 3:** Comparison of intravoxel incoherent motion parameter values and rCBV values between recurrent tumors and treatment-induced changes

	f		rCBV		D* (10^− 3^ mm^2^/s)		D (10^− 3^ mm^2^/s)	
	Mean	90th pctl	Mean	90th pctl	Mean	90th pctl	Mean	90th pctl
Tumor	0.090	0.15	3.52	4.77	11.60	29.41	1.17	1.55
Treatment-induced changes	0.058	0.14	1.79	2.53	8.81	27.45	1.15	1.44
*p*-value	0.002	0.53	0.002	0.0004	0.16	0.65	0.69	0.42

D = water diffusion coefficient, D*= pseudodiffusion coefficient, f = perfusion fraction, pctl = percentile, rCBV = relative cerebral blood volume

### Receiver operating characteristics

The ROC values for the IVIM parameters and rCBV with regard to their means and 90th percentile values are presented in Table 4. The numerically largest area under the curve (AUC) (0.79) for a single parameter was achieved by the 90th percentile value of rCBV, which also showed a little higher sensitivity and accuracy.

**Fig. 5:**
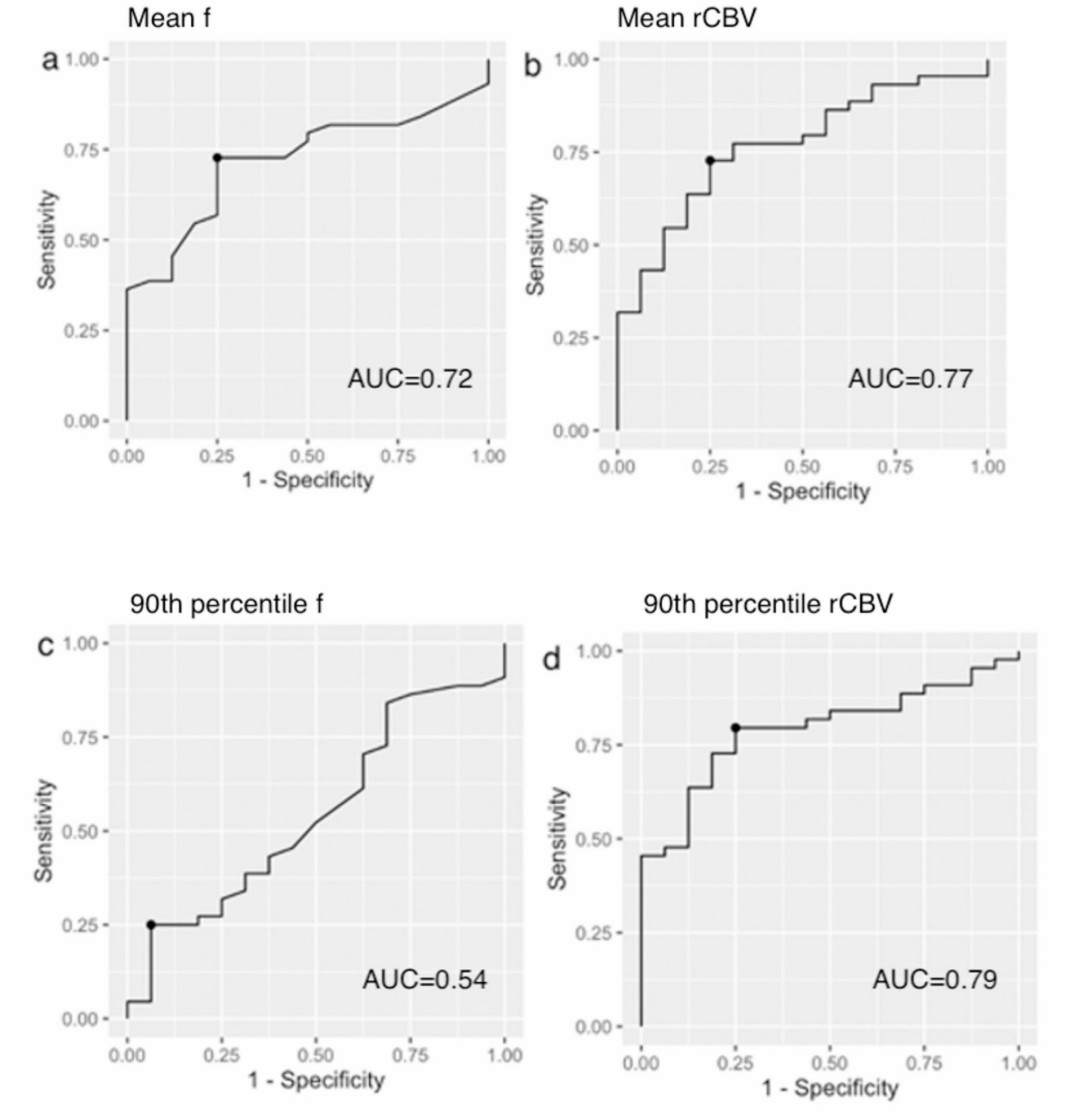
ROC curves for the means of parameters IVIM f (**a**) and rCBV (**b**); 90th percentile value of f (**c**) and rCBV (**d**). The dots on each curve represent cutoff values maximizing the sum of sensitivity + specificity. Numerically largest area under the curve of 0.79 is seen in (**d**) AUC = area under the curve, f = perfusion fraction, IVIM = intravoxel incoherent motion, rCBV = relative cerebral blood volume

A selection of ROC curves for the parameters described in Table 4 is given in Fig. 5. Cutoff values of 2.26 for mean rCBV and 0.073 for mean f yielded equal values for sensitivity (73%), specificity (75%), and accuracy (73%) (Fig. 5a–b), whereas the 90th percentile value of f had lower values.

**Table 4:** ROC values for the IVIM parameters and rCBV

^Parameter^ _Statistic_	f		rCBV		D* (10^− 3^ mm^2^/s)		D (10^− 3^ mm^2^/s)	
	Mean	90th pctl	Mean	90th pctl	Mean	90th pctl	Mean	90th pctl
AUC	0.72	0.54	0.77	0.79	0.65	0.55	0.51	0.46
Cut-off value	0.073	0.20	2.26	3.25	8.99	26.90	1.38	1.78
Sensitivity	73%	25%	73%	80%	64%	61%	18%	31%
Specificity	75%	94%	75%	75%	75%	56%	100%	75%
Accuracy	73%	43%	73%	78%	67%	60%	40%	63%

D = water diffusion coefficient, D*= pseudodiffusion coefficient, f = perfusion fraction, IVIM = intravoxel incoherent motion, pctl = percentile, rCBV = relative cerebral blood volume, ROC = receiver operating characteristics

## Discussion

We estimated perfusion values via IVIM parameters and rCBV to differentiate tumor recurrence from treatment-induced changes using measurements in the hotspots if detected, and in the entire contrast enhancements in the rest of cases. Hotspots were found in about one-half of the cases in both f and rCBV maps. We found higher interobserver and intraobserver agreements for rCBV than for f. Using both techniques, a significantly higher perfusion was observed in tumor recurrences than in treatment-induced changes, although with a substantial overlap. A similar differential diagnostic value was achieved for the mean values of f and rCBV, but accuracy was increased from 73 to 78% when the 90th percentile value of rCBV was used instead. D and D* were not useful IVIM parameters in this differential diagnosis.

We found a moderate correlation between the mean values of f and rCBV. The Pearson’s r correlation coefficient was 0.45 in the whole material and 0.50 after exclusion of an outlier. Two earlier studies that included both high- and low-grade gliomas also showed moderate correlations, Pearson’s r 0.53 and 0.59 [15, 16]. In a study of healthy subjects, a moderate correlation was found in gray matter (*r* = 0.48), but in white matter, Pearson’s r was as low as 0.02 [17]. Correlations in these previous studies as well as in our study are not particularly high, perhaps because f and rCBV reflect different parts of the vasculature. f depends on the microvascular network and rCBV is also influenced by larger vessels.

There is widespread use of rCBV to differentiate between tumor recurrence and radiation injury. In a meta-analysis of studies using rCBV for that differential diagnosis, the pooled sensitivity and specificity were 0.88 (95% CI 0.82–0.92) and 0.85 (95% CI 0.68–0.93), respectively [18]. The corresponding values were 0.73 and 0.75 in our study. The cutoff values in the meta-analysis ranged from 0.71 to 3.69, with a mean of 1.62. We found the optimal cutoff value for mean rCBV to be 2.26. A recent review on the differential diagnosis between tumor recurrence and radiation effects noted that the data regarding appropriate cutoff values for rCBV are inconsistent [19]. Differences in equipment and in imaging and analyzing methodology as well as types of pathological processes may influence the results.

In the current study, the sensitivity and specificity were exactly the same for mean f and rCBV. In an earlier study on differentiation between tumor progression and radiation necrosis that only used f for perfusion evaluation, the sensitivity and specificity were higher (1.00 and 0.80, respectively) than in our study, but only 10 cases were included and all neoplasms were metastases [11].

Some research groups have reported good results in the differential diagnosis between recurrent tumor and treatment effects when the 90th percentile value for f or rCBV is used [10–12]. However, these groups did not compare their results directly to mean f or mean rCBV, and histopathological confirmation was not obtained in all studies. In two of the studies, all treated tumors were metastases, whereas in our study, the majority of tumors were gliomas. In our study, the 90th percentile value of f did not differ between the recurrent tumors and the treatment-induced changes. The AUC for the 90th percentile value of f was 0.54 vs. 0.72 for mean f. For rCBV, the corresponding AUCs were 0.79 and 0.77. One factor affecting the results may be that we only measured in the area with the highest perfusion, if such an area existed, whereas the whole enhancing area was included in previous studies.

We found no significant difference in D* or D between recurrent tumors and treatment-induced changes. In contrast, some previous studies have showed that diffusion-weighted imaging can be useful in this setting and, in a meta-analysis on this topic, a pooled sensitivity of 71% and specificity of 87% for differentiating glioma recurrence from treatment-induced changes were reported using apparent diffusion coefficients [20].

One inherent difficulty when studying tumor recurrence is that most high-grade tumors will recur. Thus, the question is whether only treatment effects were present in our patients who later developed tumor recurrence. Another difficulty is that many lesions consist of a mixture of treatment-induced changes and tumor cells. This can also cause difficulties for the pathologist in making a diagnosis [21]. Only about one-third of our diagnoses were based on tumor specimens, which is a limitation of the study. Our intention was to collect a material reflecting the clinical reality. Therefore, we included all patients who had a new or enlarging contrast-enhancing lesion in an earlier irradiated region. The mix of tumors surely influence the results but the variety of tumors is the situation which we meet at everyday clinical work. All patients have previously been treated with radiation therapy and about half of them had received treatment with temozolomide. Temozolomide can make the treatment-induced changes occur more frequently [2], but to our knowledge the perfusion values should not differ depending on the genesis of the treatment-related changes. Our use of hotspots was an attempt to minimize the heterogeneity of the lesions. In a study of breast lesions, measurements were made in both hotspots and whole lesions, with the use of IVIM hotspots improving results in some diagnostic groups [22]. We did not undertake measurements in the whole enhancing area in all study subjects and could therefore not make the same comparison in our study. One limitation for the interpretation of the results in our study was that there was only fair to moderate agreement regarding whether a hotspot was present or not for the perfusion factor f, but the results were the same as those for the rCBV hotspots. When assessing the correlation between f and rCBV, the highest values of f and rCBV do not need to be from the exact same spatial location since the two methods are based on partly different physiological phenomena. Thus, the correct location of the recurrence may have been found by only one of the methods. In this study, lesions could be of any size, and the result in small hotspots could be greatly affected by noise. The hotspot found by the observers could come from different places in the lesion. The IVIM and DSC sequences had somewhat different spatial resolutions, with in-plane resolutions of 1.2 × 1.2 mm and 1.8 × 1.8 mm, respectively. From this follows slightly larger partial volume effects for the DSC sequence. Theoretically, this could cause a lower possibility to detect a very small hotspot, using the DSC technique. However, in our material, the hotspots measured at least 7 pixels, why we do not believe this difference has affected our comparison. The measurement result could also be affected by non-physiological values, which is the reason why we omitted f values > 0.3, as others have also done [15, 16, 23]. In this work, we used a commercially available software for calculation of the IVIM parameters. It should be acknowledged that there are other algorithms available [6], and that results may differ depending on used method [24]. Future standardization of both sequence parameters and methods for extraction of IVIM parameters would improve reproducibility and enable more accurate comparisons between different sites. Differences in T2, mostly because related to water content, between different compartments may affect IVIM estimates. To account for this, measurements at different echo times may be collected, as described in [25]. One source of error in the estimation of perfusion using the IVIM technique is that differences in water content influences values for f, since this is a proportion of the MR visible proton pool. Thus, it cannot be excluded that differences in water content between tumor recurrence and radiation reaction has affected our results. There are also other possible sources of error to take into account. To get the correct IVIM parameters the effects of noise needs to be properly handled. At high b-values the signal-to-noise ratio becomes low and the signal attenuation appears curved. This can result in underestimation of the IVIM parameter f, which can even become “negative”. The possible effect of water exchange on the estimation of IVIM parameters remains also still to be investigated [6].

A further limitation of IVIM is that a bi-exponential signal attenuation model may not be the best fit for the data in heterogeneous tissue, especially at very high b values, where non-Gaussian models better explain signal attenuation [6]. The contralateral ROI was placed in a symmetrical position. Most often that implicated a placement in normal-appearing white matter because the tumors most often originated from white matter. An alternative approach would have been to place the contralateral ROI at a predetermined normal-appearing white matter area but the location of the ROI may affect the measured signal intensity.

## Conclusion

The IVIM parameter f and the rCBV have a similar capability to differentiate tumor recurrence from treatment-induced changes. There is a moderate positive correlation between the mean values of f and rCBV. For patients that cannot be examined using contrast agents, IVIM imaging is a potential option.

## Electronic supplementary material

Below is the link to the electronic supplementary material.

Supplementary Material 1
