# Supplementary material for: Intravoxel incoherent motion imaging and dynamic susceptibility contrast perfusion MRI in differentiation between recurrent intracranial tumor and treatment-induced changes
Source: Neuroradiology. 2025 Mar 21;67(6):1423–33. doi: 10.1007/s00234-025-03575-4 (PMC12357801; doi:10.1007/s00234-025-03575-4)
Supplement: Supplementary file 1 — Supplementary Material 1 [file 234_2025_3575_MOESM1_ESM.pdf]

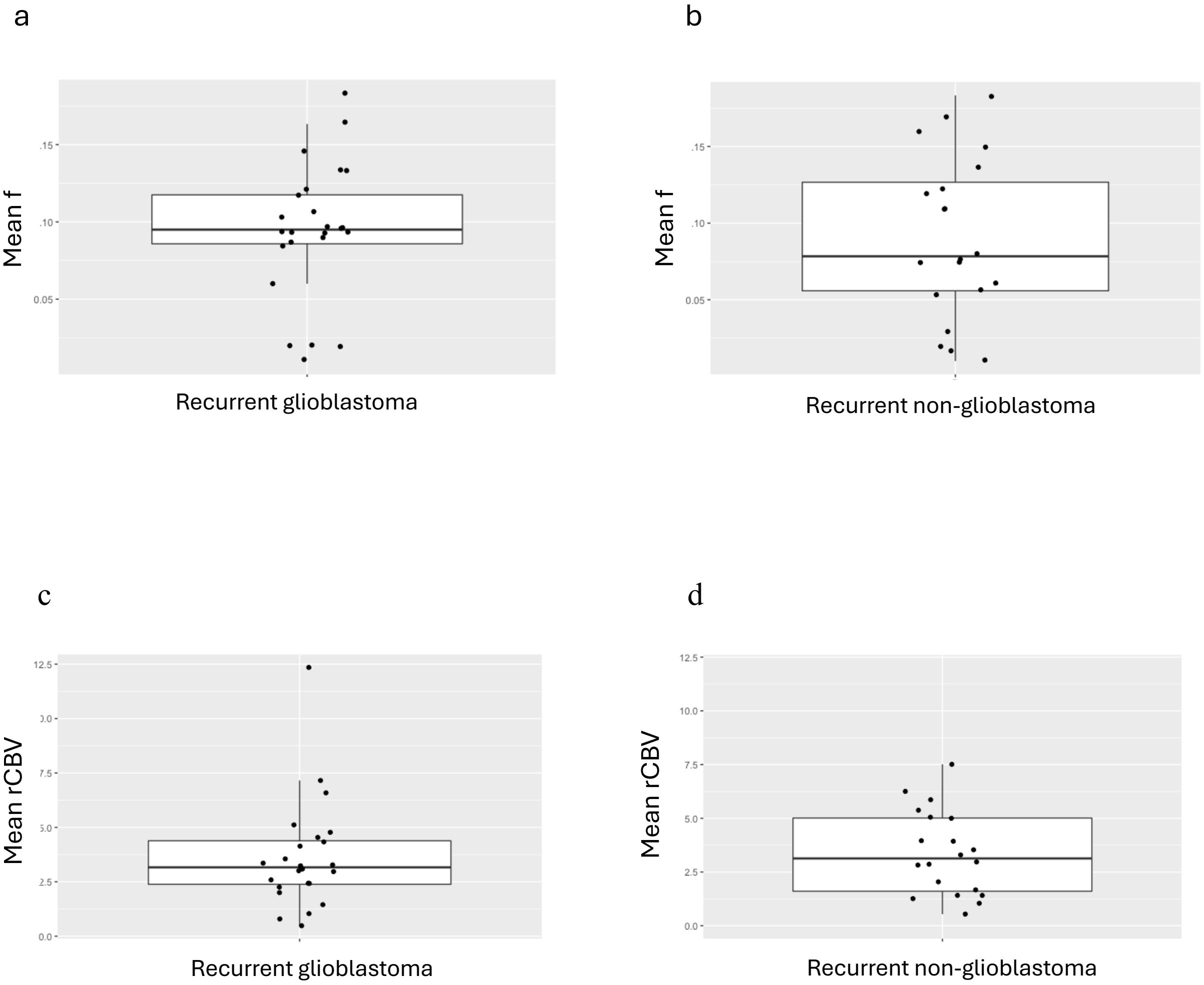

Fig. (ESM1) Boxplots with overlaid individual values in recurrent tumors where the primary tumor had been glioblastoma (a,c) and non-glioblastoma (b,d). The horizontal line in the boxes represents the median value. The whiskers represent values within 1.5 x interquartile range value above or below the edges of the box.

	Min.		1st quantile		Median		Mean		3rd quantile		Max.	
	f	rCBV	f	rCBV	f	rCBV	f	rCBV	f	rCBV	f	rCBV
Recurrent glioblastoma	0.01	0.48	0.086	2.39	0.095	3.16	0.094	3.62	0.12	4.38	0.18	12.35
Recurrent non-glioblastoma	0.01	0.54	0.056	1.61	0.078	3.13	0.091	3.39	0.13	5.01	0.18	7.51

Table (ESM1). Table showing the values of IVIM parameter f and rCBV in the recurrent tumors with primary tumor glioblastoma (n=24) and primary tumor non-glioblastoma (n=20).
